# Determinants of Overall and Readmission-Free Survival in Patients with Metastatic Epidural Spinal Cord Compression †

**DOI:** 10.3390/cancers16244248

**Published:** 2024-12-20

**Authors:** Mirza Pojskić, Benjamin Saß, Miriam H. A. Bopp, Sebastian Wilke, Christopher Nimsky

**Affiliations:** 1 Department of Neurosurgery, University of Marburg, 35037 Marburg, Germany; sassb@med.uni-marburg.de (B.S.); bauermi@med.uni-marburg.de (M.H.A.B.); wilkese@students.uni-marburg.de (S.W.); nimsky@med.uni-marburg.de (C.N.); 2 Center for Mind, Brain and Behavior (CMBB), 35043 Marburg, Germany

**Keywords:** spine metastasis, spine surgery, metastatic epidural spinal cord compression (MESCC), overall survival, readmission free survival

## Abstract

Background. The aim of this study was to assess the surgical outcomes and survival of patients surgically treated for metastatic epidural spinal cord compression (MESCC), with a specific focus on identifying factors that influence overall survival and readmission-free survival. Methods. All patients who underwent surgery for spine metastases at our department in the period 2018–2022 were included in the study. Results. A total of 175 patients (n = 71 females, median age 67.15 years) were included. The most common primary tumors were lung carcinoma (n = 31), prostate carcinoma (n = 31), breast carcinoma (n = 28), multiple myeloma (n = 25), and renal cell carcinoma (n = 11). ECOG performance status was 0 (n = 7), 1 (n = 97), 2 (n = 27), 3 (n = 17), and 4 (n = 27). Pathological fractures were present in n = 108 patients. Decompression only was performed in n = 42, additional instrumentation in n = 133, and vertebral body replacement in n = 23. The most common complications were wound healing deficits and hardware failure. Preoperative motor deficits were present in n = 89 patients. Postoperatively, n = 122 improved, n = 43 was unchanged, and n = 10 deteriorated. Mean overall survival (OS) was 239.2 days, with a 30-day mortality rate of 18.3%. Favorable prognostic factors included Tomita score < 7, Frankel score A–C, ECOG 0–1, and Modified Tokuhashi score > 10 (*p* < 0.01). Factors affecting OS and readmission-free survival (RFS) included prognostic scores, adjuvant therapy, ASA classification, surgical complications, metastasis number, and postoperative improvement. Better prognostic scores, adjuvant therapy, and clinical improvement were associated with longer OS and RFS, while complications or deterioration resulted in worse outcomes. Conclusions. Patients undergoing decompression and/or stabilization for metastatic spinal tumors showed improved outcomes, with favorable prognosis linked to Tomita score < 7, Frankel score A–C, ECOG 0–1, and Modified Tokuhashi score > 10.

## Simple Summary

Due to advancements in cancer-specific therapies and the resulting extension of survival in cancer patients, the incidence of spinal metastases has been increasing. Surgical treatment has become more important, thanks to improvements in surgical techniques and the prolonged overall survival of patients, particularly in cases of metastatic epidural spinal cord compression (MESCC), spinal instability, and intractable pain. Although several scoring systems are available to predict prognosis and outcomes for patients undergoing surgery for spinal metastases, growing evidence suggests that these systems may underestimate actual survival expectations. This underestimation may limit the potential benefits of surgery for patients who are predicted to have shorter survival and worse outcomes. In this retrospective study of surgically treated patients with MESCC, we investigated the prognostic factors that influence overall survival, readmission-free survival, as well as clinical improvements such as reversal of neurological deficits and pain reduction.

## 1. Introduction

Due to the improvement in cancer-specific therapies and the prolongation of survival of cancer patients, the incidence of spinal metastases is on the rise [1,2]. The role of surgical therapy has gained importance due to the development of surgical techniques and prolonged overall survival of cancer patients, especially in cases of metastatic epidural spinal cord compression (MESCC), spinal instability, and intractable pain [1,2]. Estimation of instability of the spine in the context of tumor burden is widely performed with Spinal Instability Neoplastic Score (SINS) [3], whereas two important factors of this scoring system such as the collapse of the vertebral body as well as the involvement of the posterior and lateral elements of the spine—which almost always lead to compression of the dural sac—predispose patients to MESCC as suitable candidates for surgical treatment. A recent study has shown that SINS correlates to the grade of MESCC and that higher SINS is a risk indicator for the development of ESCC [4]. As the spine is the most common location of skeletal metastases [5], MESCC and resulting neurological deficits and pain are a significant burden in the oncological treatment of these patients and affect severely mobilization, quality of life, and general condition [6,7]. Timely diagnosis and treatment of these lesions can reverse the deficits and maintain or improve the patient’s general condition, make them suitable for adjuvant therapies, and prolong the survival [6,7].

There is a variety of scoring systems that predict prognosis and outcomes for patients undergoing surgery for spinal metastases, with the Tomita score [8] and Tokuhashi score [9,10] being widely accepted. Although there are several prognostic scores that predict the overall survival of patients with spinal metastases, there is a growing body of literature suggesting that there is an underestimation of actual expectancy, which then limits patients with supposed shorter predicted survival and worse prognosis to profit from the surgical therapy [11]. Several known factors that have shown correlation to shortened survival are impaired functional status usually expressed through Karnofsky Performance Score (KPS), Eastern Cooperative Oncology Group Score (ECOG) [12], or through the American Society of Anesthesiologists (ASA) score in terms of suitability for surgery, as well as the presence of metastases other than in the spine, advanced age of 65 years and older, and the presence of multiple spinal metastases [13]. Functional status and ambulation of patients can be indirectly expressed also through preoperative and postoperative American Spinal Injury Association (ASIA) score [14] and Frankel grade [15]. Historically, the success of surgery for MESCC was deemed by complications and mortality rate in the short postoperative period, usually in the first 30 days [16]. Overall survival (OS) is one of the most common indicators of patient status. Recently, an oncological concept of significant importance in evaluating treatment success, known as readmission-free survival (RFS), has emerged. RFS measures the time from the primary treatment to the patient’s readmission due to disease progression [16,17].

This study addresses the limited and inconsistent data on factors influencing survival outcomes in MESCC patients, particularly regarding RFS. By examining the determinants of both OS and RFS, the study aims to fill these gaps and improve predictive accuracy. Identifying key clinical and treatment factors associated with prolonged OS and RFS will provide valuable insights into patient prognosis. The findings may guide clinical decision-making, optimizing treatment strategies and potentially improving survival and quality of life by reducing readmission rates in MESCC patients.

## 2. Material and Methods

### 2.1. Study Design and Patients

The research was designed as a retrospective observational study conducted at the Department of Neurosurgery, University Hospital Marburg. All patients who were surgically treated for MESCC from January 2018 to December 2022 were included in the study. A total of 175 consecutive patients with a confirmed diagnosis of metastatic spinal disease with epidural compression who were surgically treated were included in the study. Inclusion criteria comprised: (a) patients aged 18 years or older, (b) patients with confirmed primary tumor as the source of metastasis, (c) patients who underwent decompression surgery and/or stabilization and/or reconstruction with expandable cage following corpectomy for spinal metastases during the specified period. Patients who did not meet the criteria were excluded from the study. Ethical approval was obtained from the Ethics Committee of the University Hospital Marburg on the 9th of November 2022, under reference number RS 22/73, which deemed ethical approval for this retrospective study unnecessary.

### 2.2. Methods

#### 2.2.1. Data Extraction

Patient data were extracted from the hospital’s information system and included sociodemographic characteristics, localization of the primary tumor, affected topographic regions of the spine, number of metastases, extent of tumor, operative strategy derived from operative reports, perioperative modality of treatment as well as perioperative complications.

#### 2.2.2. Patient Evaluation and Follow-Up

Before the surgical intervention, ECOG score was determined, classifying patients into four groups: 0—asymptomatic, 1—symptomatic but completely ambulatory, 2—symptomatic but <50% in bed during the day, 3—symptomatic but >50% in bed during the day, 4—bedbound [18]. Patients with ECOG ≤ 2 were considered to have a milder clinical presentation, while patients with ECOG > 2 were considered to have a more severe clinical presentation according to Hess et al. [17]. For patient evaluation, the Tomita predictive score (TPS) was utilized, consisting of six variables: general condition, the number of extraspinal bone metastasis foci, number of metastases in the vertebral body, metastases to the major internal organs, the primary site of cancer, and spinal cord palsy. Each parameter has a scale from 0 to 2, with values closer to zero indicating a more favorable patient status. The total score on the scale is 12. A Tomita predictive score < 5 suggests palliative methods for surgical treatment, while a score greater than 9 recommends palliative care [8]. Based on the Tomita score he threshold value in this study is >7 [19]. The Modified Tokuhashi Score (MTS) was also utilized, with values grouped into category A (0–8 points)—predicting survival of less than six months, category B (9–12 points) with survival longer than six months but less than one year, and category C (13–15 points) with survival longer than one year [9,10].

Preoperative risk assessment for surgical intervention was conducted using the ASA classification: I–healthy patients, II—a patient with mild systemic disease, III—patients with severe systemic disease, IV—patients with severe systemic disease that is a constant threat to life [20]. Motor and sensory function impairment was assessed using the ASIA impairment scale, categorizing individuals into Grade A (complete), Grade B (sensory incomplete), Grade C (motor incomplete with sparing), Grade D (motor incomplete with significant sparing), or Grade E (normal) based on the preservation of sensory and motor functions at specific neurological levels. Further assessment of spinal cord injury was performed also using Frankel grade, with A–E classes according to the amount of spinal cord injury [15]. Additionally, the time of readmission to the department due to worsening conditions or the date of death, following discharge from initial surgery was also recorded as RFS [16]. Patients were followed up until June 2024. Time to death as an estimation of OS was estimated by either taking the date of death as an end-point or censoring patients either at their last follow-up visit if the date of death was unknown, or at two years after surgery, analog to previous studies [21]. Neurological improvement or surgical treatment effect was defined as improvement or reversal of preoperative neurological deficits, objectively expressed as a change in class in the Frankel grading system and ASIA scale. All scores were evaluated postoperatively, i.e., at the time of the last presentation of the patient at the end of the follow-up. Overall survival (OS) was defined as a time frame from surgical treatment until the death of the patient. For patients who did not die until the end of the follow-up, the timepoint of the last presentation was taken as the estimation of OS. As previously mentioned, readmission-free survival (RFS) was defined as the time from primary treatment to the patient’s readmission due to disease progression.

**Figure 1:**
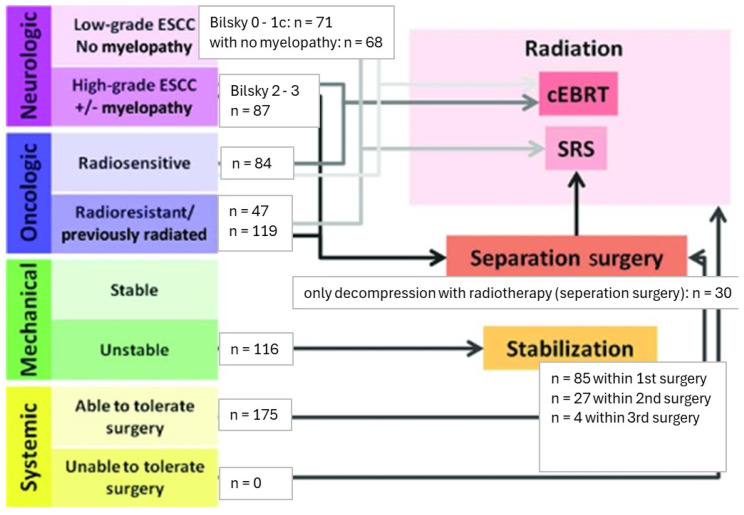
Distribution of patient cohort according to the NOMS Framework and Bilsky classification (Figure adapted from [22]).

Interdisciplinary treatment of patients with spinal metastases was performed according to recommendations of the neurologic, oncologic, mechanical, and systemic (NOMS) decision framework [22]. Figure 1 provides a classification of our cohort of surgically treated patients according to the NOMS framework. Indications for treatment type, i.e., form of surgical therapy—decompressive surgery vs. stabilization, was decided upon Bilsky classification [23] as well as spinal neoplastic instability SINS [3]. The number of patients who underwent radiation (cEBRT/SRS) only, without surgical treatment, at our university hospital could not be determined, as our data collection is based on patients who underwent surgery at our department. Figure 1. contains a parallel to the classification of patients according to the NOMS framework and also a division of the cohort according to the Bilsky score as well as a treatment modality. Patients with radiosensitive tumors were deemed as primary diagnoses of breast and prostate cancer, myeloma, and lymphoma.

### 2.3. Statistical Analysis

For statistical analysis, SPSS software (version 27.0, IBM Inc., Armonk, NY, USA) was utilized. Continuous variables were expressed as means or medians with corresponding 95% confidence intervals, while categorical variables were presented as frequencies (N) and percentages (%). The Kolmogorov–Smirnov test assessed the normality of distributions, and non-parametric methods such as the chi-square test were employed for variables exhibiting deviations from normality. The relationship between preoperative patient status and postoperative outcomes was explored through multivariate regression analysis. Additionally, overall survival (OS) time, from metastatic spinal disease diagnosis to death, was calculated, and Kaplan–Meier plots compared OS and readmission-free survival (RFS) across specific preoperative clinical status variables. Median OS and RFS, along with their 95% confidence intervals, were reported. Statistical significance was established at *p* ≤ 0.05.

## 3. Results

### 3.1. Sociodemographic Data

Table 1 presents sociodemographic characteristics of the study population, revealing that males comprised 59.4% (N = 104) while females accounted for 40.6% (N = 71) of participants (*p* = 0.013). The median age of participants was 67. Age distribution showed that 24.6% were under 59 years, 65.7% fell within the 60 to 80 years category, and 9.7% were older than 81 years (*p* < 0.001).

### 3.2. Preoperative Findings

The distribution of primary neoplasms showed significant variability (*p* < 0.001), with lung and prostate cancers being the most prevalent (17.7% each), followed by breast cancer (16.0%) and multiple myeloma (14.3%). Affected regions demonstrated diversity (*p* = 0.024), with over 45% of patients exhibiting metastases in more than two regions. Moreover, the number of metastatic lesions also exhibited significant differences (*p* = 0.011), with over 47% of patients having more than five lesions. The extent of tumor spread was significantly associated with outcomes (*p* < 0.001), with a vast majority of patients (94.3%) exhibiting epidural involvement. Furthermore, the administration of adjuvant chemotherapy (*p* < 0.001) and adjuvant radiotherapy (*p* < 0.001) significantly varied, with 52.8% and 71.7% of patients receiving them, respectively (Table 2).

Primary tumors were known in 111 patients, i.e., 63.43% of patients were already diagnosed with a primary tumor and underwent previous oncological treatment. The average time from the beginning of symptoms to diagnosis was 75 days. The time from the diagnosis of the primary tumor to MESCC or for symptoms to begin (in days) was 1123 days.

### 3.3. Clinical Presentation, Surgical Outcome, and Complications

Table 3 presents the clinical presentation, surgical outcome, and complications among patients. Significant associations were observed for various variables. In terms of neurological deficits, 122 patients improved, 43 were unchanged and 10 deteriorated. Regarding the ECOG performance status, most patients were symptomatic but completely ambulatory (55.4%), while only 4.0% were asymptomatic (*p* < 0.001). TPS exhibited significant variation (*p* < 0.001), with most patients (45.7%) falling into the > 24 months category requiring surgical strategy. Similarly, the MTS showed significant differences (*p* < 0.001), with most patients (58.3%) classified as A (<six months). The Frankel score also demonstrated significant variability (*p* < 0.001), with most patients falling into categories D and E (38.3% and 39.4%, respectively). ASA classification exhibited significant associations (*p* < 0.001), with the majority of patients classified as III (72.0%). ASIA impairment scale showed significant differences (*p* < 0.001), with the majority of patients classified as grade D (52.0%). The treatment effect demonstrated significant variability (*p* < 0.001), with the majority of patients showing improvement (69.7%).

In 108 patients, pathological fracture was present. In 42 patients only decompression was performed, in 133 additional instrumentation surgery, 23 patients underwent vertebral body replacement with an implant. Urgent surgery, i.e., in the first 12 h following the presentation was performed in 48 patients or 27.42%, whereas the rest of it were surgeries that were performed at a time scale of more than 12 h and during the regular working hours. At initial surgery, decompression only was performed in 72 cases, and decompression and stabilization in the rest of the cases. In 154 cases primary surgery was performed via dorsal approach, in 17 cases ventral, and in five cases dorsoventral. A total of 102 patients had one surgery, 46 patients had two surgeries, 16 patients had three surgeries, eight patients underwent four surgeries, two patients had five surgeries, and one patient underwent six surgeries. Stabilization surgery was performed on a total of 125 (71.4%) patients. Type of surgery and invasiveness (decompression vs. decompression + stabilization) as well as the number of secondary surgeries for stabilization did not have a significant influence on OS or ReASF (*p* < 0.01), whereas the number of surgeries due to revision was significantly correlated to OS and RSF as a complication (*p* < 0.05). Complications after surgery also significantly varied (*p* < 0.001), with wound revision being the most common complication (5.7%), while 66.9% of patients experienced no complications. The median surgery time was 151 min (IQR: 105–206) (*p* = 0.059), and the median blood loss during surgery was 500 mL (IQR: 300–800) (*p* < 0.124).

### 3.4. Association of Preoperative Findings with Surgical Outcome

Patients experiencing improvement in ECOG status exhibited a substantial increase in odds of deteriorated outcomes, with an odds ratio (OR) of 5.585 (95% CI: 1.113–28.031, *p* = 0.037), while those displaying no change also demonstrated an OR of 8.400 (95% CI: 1.480–47.667, *p* = 0.016). Conversely, no statistically significant associations were observed between outcomes and categories of the Tomita Score, Frankel Score, or Modified Tokuhashi Score (Table 4).

### 3.5. Factors Associated with Overall Survival

**Figure 2:**
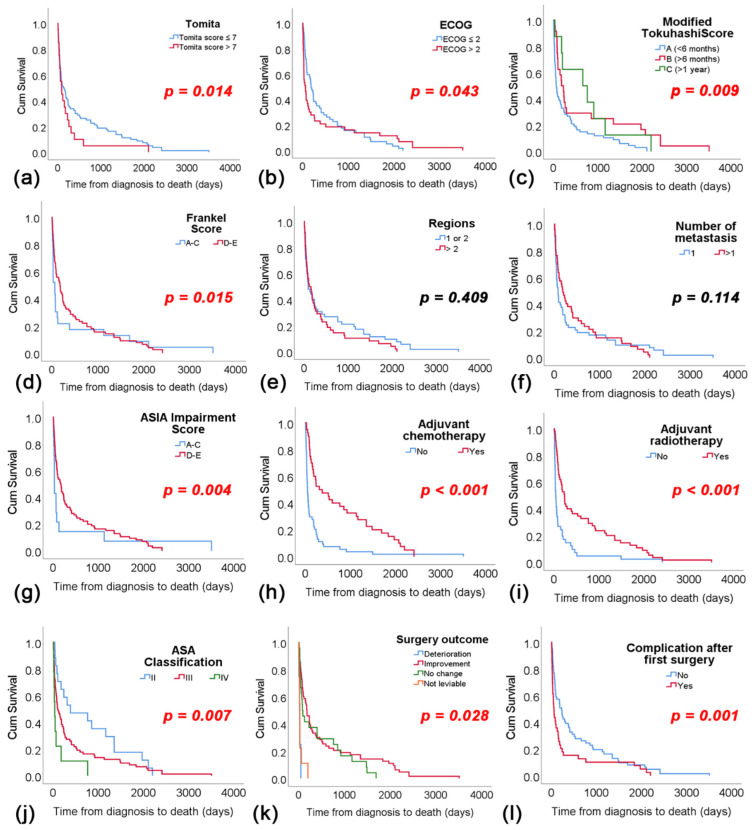
Overall survival in different Tomita (**a**), ECOG (**b**), Modified Tokuhashi (**c**) and Frankel score (**d**), as well as in comparison to different regions of the spine (**e**), number of metastases (**f**), ASIA impairment score (**g**), adjuvant chemotherapy (**h**) and adjuvant radiotherapy (**i**), ASIA classification (**j**), surgery outcome (**k**) and complications after first surgery (**l**).

The mean OS for the entire cohort was 239.2 days. The median follow-up period was 8.02 months (IQR: 8.02–22.95) among 100 patients (57.14%) with confirmed mortality during the specified period. Patients were followed up until June 2024, i.e., a minimum of 18 months following surgery (Figure 2).

In 100 patients with a known date of death, a 30-day mortality rate was noted in 32 patients out of 100 (32%). A total of 25 patients have died in up to 1–3 months following surgery, 18 patients in 3–6 months following surgery, 10 patients in 6–12 months, seven patients in 12–24 months, and eight in more than 24 months. For 75 patients, the date of death was not known, and the last presentation was taken as an estimate of OS. Since this last presentation was in all cases with an unknown date of death in more than 30 days following surgery, the overall, 30-day mortality rate was 32 patients out of 175 or 18.3%. In 75 days without a known date of death, the last presentation was in up to 1–3 months in 28 patients, in 10 patients in 3–6 months, in 12 patients in 6–12 months, in 10 patients in 12–24 months and in 15 patients in more than 24 months.

Patients with a Tomita score ≤ 7 had a median OS of 132 days (95% CI: 23.5–240.5), significantly longer than those with a Tomita score > 7 (*p* < 0.014), who had a median OS of 92 days (95% CI: 21.9–162.1). For patients with ECOG ≤ 2, the median OS was 188 days (95% CI: 115.1–260.9), significantly longer than those with ECOG > 2, whose median OS was 67 days (95% CI: 9.2–124.8) (*p* = 0.043). Concerning the Modified Tokuhashi Score, patients with a score A had a median OS of 68 days (95% CI: 42.8–93.2), significantly shorter than those with a score B, who had a median OS of 186 days (95% CI: 81.6–290.4) (*p* = 0.009), and a score C, who had a median OS of 396.0 (95% CI: 98.4–1441.1). Additionally, patients with Frankel Score categories A–C had a median OS of 67 days (95% CI: 7.5–126.5), significantly shorter than those with categories D-E, who had a median OS of 180 days (95% CI: 83.5–276.5) (*p* < 0.015).

The number of affected vertebral regions and metastatic lesions did not significantly affect OS (*p* = 0.409 and *p* = 0.114, respectively). ASIA impairment score indicated that patients with scores A–C had an OS of 30 days (95% CI: 27.6–32.4), compared to D-E scores with an OS of 167 days (95% CI: 90.7–243.3) (*p* = 0.004). Patients who received adjuvant radiotherapy had a longer OS of 217.4 days (95% CI: 41.2–738.1) compared to those who did not (OS: 49.0; 95% CI: 27.4–70.6) (*p* < 0.001). Additionally, patients undergoing adjuvant radiotherapy had a longer OS compared to those who did not (236 vs. 42 days, *p* < 0.001). Regarding preoperative ASA classification, patients with ASA class II had a significantly longer OS of 387.0 days (95% CI: 0.0–1094.2) compared to those classified as III (OS: 104 days; 95% CI: 35.2–178.3) and IV (OS: 38 days; 95% CI: 11.7–64.3) (*p* = 0.007). Surgical outcomes classified as deterioration of motor and sensory function significantly reduced OS (*p* = 0.007) to 21 days (95% CI: 3.4–38.6) compared to patients with postoperative improvement (OS: 186 days; 95% CI: 97.1–275.0). Post-surgery complications also significantly reduced OS (*p* = 0.001) to 60 days (95% CI: 34.5–85.5) compared to patients without complications (OS: 228 days; 95% CI: 156.9–229.0).

**Figure 3:**
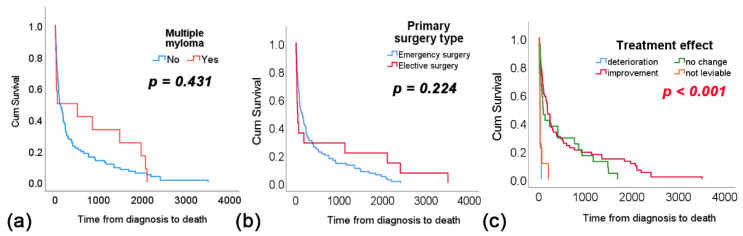
Differences in overall survival (OS) for: (**a**) multiple myeloma patients, (**b**) primary surgery type, and (**c**) treatment effect.

Given the radiosensitivity, the impact of multiple myeloma as a type of metastatic spinal tumor was analyzed, but no difference in survival time was observed (Figure 3a). The urgency of surgery did not affect survival duration (Figure 3b), whereas patients who experienced postoperative clinical improvement had significantly longer overall survival, with a median of 45 days (*p* < 0.001) (Figure 3c).

### 3.6. Factors Associated with Readmission-Free Survival

**Figure 4:**
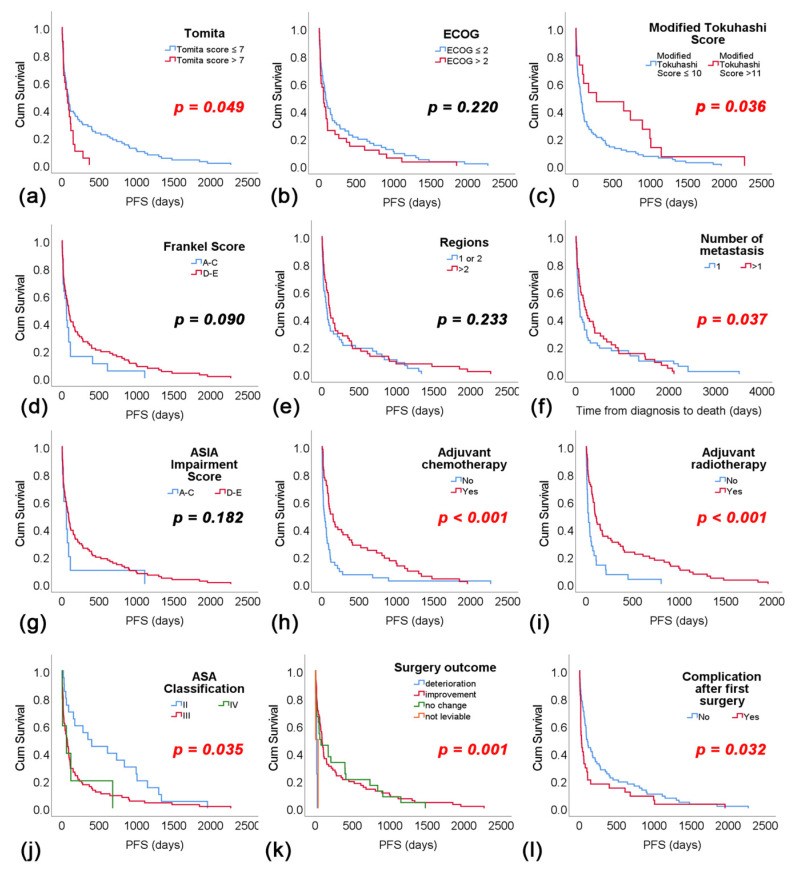
Readmission-free survival for observed variables: (**a**) Tomita score, (**b**) ECOG score, (**c**) modified Tokuhashi score, (**d**) Frankel score, (**e**) regions of the spine, (**f**) number of metastases, (**g**) ASIA impairment score, (**h**) adjuvant chemotherapy and (**i**) radiotherapy, (**j**) ASIA classification, (**k**) surgery outcome and (**l**) complications after first surgery.

Patients with ECOG score ≤ 2 exhibited a median RFS of 90 days (95% CI: 54.5–125.5), while those with ECOG score > 2 had a median RFS of 61 days (95% CI: 23.9–98.1), with a non-significant difference observed (*p* = 0.220). Conversely, patients with a Modified Tokuhashi Score > 11 demonstrated a significantly longer median RFS of 280 days (95% CI: 0.0–959.2) compared to 73 days (95% CI: 47.4–98.6) for those with a score ≤ 10 (*p* = 0.036). The average RFS was 283.74 days. No significant difference in RFS was found between patients with Frankel Score categories A–C (RFS: 61 days, 95 % CI: 28.3–93.7) and D-E (RFS: 90 days, 95 % CI: 56.3–123.7) (*p* = 0.090). The number of affected vertebral regions did not significantly affect RFS (*p* = 0.233). However, patients with more than one metastasis had a significantly shorter median RFS of 52 days (95% CI: 13.1–90.9) compared to 108 days (95% CI: 74.5–141.5) for those with one metastasis (*p* = 0.037). ASIA impairment score categories did not exhibit a significant difference in RFS (*p* = 0.182). Furthermore, patients who received adjuvant chemotherapy had a significantly longer median RFS of 151 days (95% CI: 70.5–231.5) compared to those who did not (RFS: 33 days, 95% CI: 0.5–65.5) (*p* < 0.001). Similarly, patients undergoing adjuvant radiotherapy had a significantly longer median RFS of 101 days (95% CI: 73.1–128.9) compared to those who did not (RFS: 27 days, 95% CI: 13.8–40.12) (*p* < 0.001). ASA classification showed a significant difference in RFS, with class II patients demonstrating a median survival of 350 days (95% CI: 93.6–606.4) compared to 70 days (95% CI: 41.2–98.8) for class III and 61 days (95% CI: 0.0–174.8) for class IV (*p* = 0.035). Lastly, surgical outcome significantly influenced RFS, with patients experiencing improvement showing a median survival of 91 days (95% CI: 66.1–115.9) compared to 18 days (95% CI: 3.3–32.7) for those experiencing deterioration (*p* = 0.001) (Figure 4).

## 4. Discussion

A recent epidemiological study which has included 56 studies revealed that 10% of all patients with spinal metastases will develop MESCC and a further 12.6% pathological fracture, with a significant delay in diagnosis and beginning of the symptoms [24]. Improvement of symptoms in patients with MESCC is best achieved through surgery than with radiation therapy [25], which is in cases of radioresistant tumors and cases of severe spinal canal stenosis and the presence of neurological deficits contraindicated as primary therapy. MESCC with severe pain, instability of the spine, and progressive neurological deficits are all indications for operative treatment [26], whereas presumable life expectancy should be in ideal case from 3 to 6 months [26] but not less than 2–3 months [27,28]. However, the question of maintaining the quality of life has emerged in the past two decades and there are opinions that surgical therapy should be performed also in patients with reduced expected survival and poor performance status. A recent study has shown that in comparison between patients who lived less than three months had a quality of life in six weeks after surgery comparable with those who lived longer and that this variable is independent of survival [29]. Palliative surgery for MESCC in all patients with intractable pain as well as with high-grade motor neurological deficits, where timely surgery can lead to their reversal and enable mobilization and ambulation of the patient, is a good example of proper indication for surgery, regardless of performance status at the presentation and estimated prognosis.

A favorable prognosis in this study was shown in patients with Tomita score < 7, Frankel score A–C, ECOG 0–1, and modified Tokuhashi score > 10. Poor baseline (ECOG 3–4) has shown a correlation to readmissions, reduced OS, and early reoperations [30]. Although there are several large studies that examine the role of prognostic factors in surgically treated spinal metastases, evidence has been shown to be very low for most of the predictors [31]. A recent prospective multicenter cohort of 1430 patients with spinal metastases has shown that the cause of death was directly due to the tumor in 87.6% of patients but in only 0.9% due to surgical complications [21]. Correlation between the preoperative neurological status of the patients and OS was shown constantly throughout the literature [32]. Whereas tumor type, the number of spinal metastases, and the presence of visceral metastases predict survival, a multicenter cohort study has shown that Karnofsky, Frankel, and EQ-5D (European Quality of Life 5 Dimensions) scores are useful for prediction of quality of life [33]. Our results are consistent with the current literature. The predictive value of Tomita [21,34] and Tokuhashi score [21,34,35,36] as well as the estimation of the general condition through Karnofsky [34,37] or ECOG [13,36] have been shown to predict OS, RSF, and six months mortality, even in patients who present with impending deficits and require acute surgical treatment [38]. Modified Tokuhashi score and Frankel score have been shown to predict postoperative neurological recovery [39]. A cut-off value for Karnofsky has been demonstrated to lie in >50% for improved outcomes, whereas patients with <40% show a high risk of unfavorable outcomes [37]. Preoperative functional status and neurological status, expressed through Frankel grade and ASIA impairment score, have shown consistent correlation to favorable outcomes [13,21,33,34,40].

Prediction of life expectancy in oncological patients is getting more complex by the day, not only due to the diversification of categories such as primary histology into multiple subtypes between the same organ tumor due to a variety of emerging genetic and molecular biology traits, which are potential targets for chemotherapy or immune therapy [41]. Although multicentric studies, which involved 1469 patients [42], have shown that the two traditional scoring systems—Tokuhashi and Tomita—have a low precision ranging from 33% to 64%, inferior to the Bollen classification [43], probably due to the fact that these systems do not incorporate the entire newly emerged genetic and molecular landscape of biomarkers, both of the systems are established in clinical practice and widely used in surgical studies. However, the use of these scoring systems has become more of a descriptive one, since the decisions on individual surgical and adjuvant treatment of the patients are increasingly being made according to symptoms and with the goal of preservation of quality of life, often regardless of general outcome prediction. Tomita score, which takes primary tumor location, presence of visceral metastases, and presence of bony metastases into consideration and does not suggest surgery for patients with worse prognosis and 8–10 points, is becoming slowly obsolete for treatment planning. The newly proposed “shiny” model has shown high consistency between predicted and actual survival, taking into consideration primary tumor and site of metastasis, visceral metastasis, Frankel grade, operation category, number of surgical segments, and the preoperative percentage of lymphocyte [7]. This study with 185 patients differs between three types of surgical treatment: posterior decompression in 60% of patients, subtotal tumorectomy in 33.5%, and the rest with en bloc spondylectomy, and a number of patients with stabilization surgery of 83%. The New England Spinal Metastasis Score (NESMS) for prediction of 30-day mortality and major complications was shown to be superior to Tomita, Tokuhashi, and SINS in a retrospective analysis of 776 cases [44]. Recently, the role of postoperative early rehabilitation through enhanced recovery after surgery (ERAS) programs has been shown to lead to earlier mobilization, removal of urinary catheters, and decreased use of opioids [45].

Advanced age has been traditionally linked to worse prognosis and reduced survival [46]. However, a retrospective study that included 412 patients at a single center did not find differences in complication rates between patients younger and older than 70 years, with almost 60% of patients in the older group who have outlived their prognosis [47]. This aspect is especially important in aging societies, with a large number of patients who are older than 80 years. When comparing these patients with groups of 60 to 70 and 70 to 80 years old, a Japanese study has shown that although initial improvement is seen throughout the groups, the rate of complications and re-deterioration is higher in patients older than 80 [48]. Lower recovery rate in patients older than 80 years, as well as a higher complication rate, was also shown in a multicenter prospective study by the Global Spine Tumor Study Group (GSTSG) with 1266 patients [49], yet due to improved quality of life, this study suggests that the surgeons should not be biased against treating these patients and that the age is not a contraindication for surgery. Yonezawa et al. also advocate surgery for older patients in spite of the high complication rate, especially in cases of renal and thyroid cancer [50]. There were no differences in gender with respect to OS and ReASF. Among 17 parameters of poor outcome for surgically treated spinal metastases, identified by Luksanapruksa et al. [30], the male gender was one of them. Further risk factors include preoperative neurological deficit with non-ambulation, multiple bone and spinal metastases as well as development of deficits shortly prior to radiation therapy, among others [30]. Similar results were shown by Goodwin et al. [51], where female patients were shown to have fewer complications and longer survival, although both groups had a quality-of-life improvement following surgery. Advanced age has also been shown in contemporary studies to influence OS in terms of 180-day mortality rate, next to the presence of extraspinal metastases, and ECOG [13]. Recent literature review and meta-analysis by Bresolin et al. [52] showed an improvement rate in terms of motor neurological deficits in 83% of all patients who underwent surgery for MESCC in the first 48 h following presentation, with a complication rate between 25.5% (<24 h surgery) and 36.8% (24–48 h). Compared to late surgery (>48 h following onset of symptoms), patients who underwent early emergent or urgent surgery have shown improved clinical outcomes and survival.

Several studies have examined the relationship between the type of surgery and outcome in patients with spinal metastases. Literature shows confounding data. One systematic review has shown that the overall complication rate seems to be lower in open cases, whereas the incidence of major complications seems to be lower in minimally invasive surgery (MIS) compared to open surgery, with a similar rate of neurological recovery [53]. One further review on stabilization surgery in the thoracolumbar spine concluded that percutaneous surgery in comparison to open shows a lower complication rate, less blood loss, and fewer infections [54]. This review, however, did not include only patients with MESCC, which usually require open surgery for decompression. Although lesions of the cervicothoracic junction were identified as a possible region of the spine that is predictive for poor outcomes and higher risk of complications [36], a 30-day complication rate has shown no differences in relation to the region of the spine in a Canadian retrospective study including 191 patients [35]. The thoracic spine has shown to be the most prevalent region for acute surgery [55] with a higher improvement rate in patients who are treated with early surgery < 16 h following admission [55]. The role of preoperative body mass index (BMI) remains unclear since a multicenter study of the German Spine Registry (DWG) has failed to demonstrate differences between obese and non-obese patients [56], in contrast to preoperative general condition and suitability for surgery expressed through ASA score, which has shown significant impact on complication rate using the data of the same registry [57]. Prognostic nutritional index (PNI) did show association with 9- and 12-month mortality in a recent study, regardless of ECOG [58]. Blood loss of more than 500 mL and transfusions have been shown to increase the likelihood of cerebrovascular events and pulmonary insufficiency. A retrospective review of 318 patients who underwent surgery for MESCC as a single procedure with decompression and instrumentation reported a hardware failure rate of 2.8%, with a significant increase when a number of instrumented levels was larger than six [59]. Frailty was predictable of surgical complications, with a higher incidence in the lumbar and junctional spine [60].

The complication rate has been generally reported as being high from 12.2% [61], 16.5% [57], 16.8% [7] 22% [1]. Reported systemic complications include pulmonary embolism, deep venous thrombosis, vascular events, or vertebral fracture [61]. Wound-related complications and neurological deterioration are reported in up to 24% of patients in contemporary surgical cohorts [13]. Strategies for reduction in wound-healing deficits include reduction in wound surface through the employment of MIS, which in the context of MESCC is usually not an optimal surgical strategy, and application of topical vancomycin, although randomized controlled trials have shown that it might not have a protective effect but instead increase the infection rate with gram-negative bacteria [62]. In a mixed cohort of 197 patients with and without MESCC, vertebroplasty has shown the lowest revision rates followed by decompression alone, with the highest rates in patients who underwent stabilization [63]. Further single-center study with 331 patients with spinal metastases has shown an increased complication rate in instrumented patients, without differences in neurological outcomes [64]. Asymptomatic construct failure has been reported in 16.7%, with predominance for junctional regions [65].

The role of radiotherapy in MESCC patients was examined in several studies. Although patients with a neurological impairment that disables walking profit more from decompression followed by radiotherapy than from radiotherapy alone [6], treatment of ambulatory patients with MESCC seems to alleviate these differences in terms of local control and ambulatory function; however, surgery leads to improved quality of life [66]. A systemic review has shown a trend of improved local control with stereotactic body radiotherapy (SBRT) compared with conventional external beam radiotherapy (EBRT) [66]. One possible negative effect of radiotherapy on wound healing was not proven in a recent study on 250 patients, regardless of whether radiotherapy was performed preoperatively or postoperatively and regardless of the modality of radiotherapy [67]. The absence of preoperative radiation did show a correlation with improved quality of life at twelve months following surgery in a recent study on 150 patients [68]. In a small sample of 104 patients, preoperative radiation and mechanical instability have been shown to negatively impact survival [69]. Adjuvant, postoperative radiotherapy, and chemotherapy have been shown to improve OS [32]. Chanbour et al. [70] report improved the 1-year survival in patients who underwent early radiation in 1–3 months following surgery. Postoperative radiotherapy has shown reduced wound-healing problems compared to preoperative in a systemic review, with an interval of seven days to two weeks as an optimum for initiation of radiation therapy following surgery [71].

Improved OS was shown in patients with Tomita score ≤ 7, ECOG ≤ 2, ASA score of II, Modified Tokuhashi Score of more than 10, and ASIA Scores A–C as well as in patients with adjuvant radiotherapy. Patients with postoperative neurological deterioration and patients with postoperative complications had shortened OS. A modified Tokuhashi score has shown better accuracy in predicting the actual survival, as a retrospective study has shown, in comparison to the Tomita score [72]. OS is usually shorter in patients who have MESCC and primary tumors with worse prognoses compared to tumors with improved potential for adjuvant treatment modalities such as breast cancer [21,73]. A systematic review has shown that the median OS was highest for breast and renal cancers and lowest for prostate and lung cancers [73]. The influence of primary tumor histology in our cohort was also shown by the positive prognostic value of a modified Tokuhashi score on OS and ReASF. In this scoring system, breast and prostate cancer carry five points and renal cancer carries three points, but lung cancer with the worst prognosis carries only zero points [9,10]. As for the further visceral and brain metastases, this was shown to be associated with a shortened OS in patients with the so-called unfavorable clinical profile, i.e., cancer types with worse prognosis [43]. Development of immunotherapy and targeted therapy with, for example, cytokines have led to a rapid increase in life expectancy for certain tumor types. Recent population studies have shown the longest OS for thyroid breast and melanoma primaries, with a 1-year mortality rate of 59% [61]. Median OS was reported to be 222 days [40], 236 days [61] 7.5 months [13], 10 months [4], and 501 days [74]. A total of a 30-day mortality rate was reported from 2.6% [75], 8.1% [61], 8.7% [76] to 17% [77] with male sex, emergency surgeries, and tumor type, as well as comorbidities [77] being the most important prognostic factors [75]. Differences in comparison to our study can be partially explained by the fact that our study has included only patients with MESCC who underwent decompression surgery and a high number of patients with instability who required secondary surgery, which has all postponed initiation of adjuvant treatment, as well as with high median age at the time of surgery, which poses itself a risk factor for reduced OS. One further reason can be the high incidence of patients with primary tumors that carry unfavorable prognoses, such as lung cancer. Subgroups of patients with favorable primary tumor histology, such as breast cancer, have shown OS with up to 32.2 months [78]. The presence of liver and lung metastases shows reduced OS [21] as well as a number of spinal metastases [21].

An improved ReASF was significant in patients with Modified Tokuhashi Score > 11, Frankel Score D–E, in patients with metastasis in one region of the spine, in patients with adjuvant radiotherapy and chemotherapy and in patients with ASA II, and with neurological improvement following surgery. In contrast to the study of Madhu et al. [79], ECOG score and primary tumor histology did not show a significant difference with respect to ReASF, yet the absence of complications and unchanged or improved neurological condition have also shown a correlation to RSF. Further important parameters, which were pointed out by Madhu et al., are preoperative hemoglobin (Hb) level > 12 g/dL as well as a short index stay and less than three comorbidities. Hb level was not assessed in this study, due to a significant number of patients who had multiple surgeries. There has recently been an initiative to differentiate between short-term 90 days and long-term ReASF, whereas patient- and treatment characteristics have been shown to influence the first one and histology-related characteristics of the primary tumor the latter [79]. Comorbidities are a known predictor of readmission [80] and the cut-off lies somewhere between three and four comorbidities [79]. This points out the importance of risk stratification when planning surgical therapy for these patients. Proposed strategies to avoid unplanned readmission include preoperative blood transfusion management, perioperative embolization, employment of minimally invasive surgery for reduction in wound surface and infection risk, use of stereotactic radiation body therapy, and other modern techniques which alleviate the need for very invasive maximal tumor resections but shift the strategy more into less invasive separation surgery, as well as early initiation of rehabilitation with respect to the stability of the spine [16,71,74,81,82,83]. Employment of percutaneous screws and minimally invasive spine surgery (MISS) was advocated, since studies have shown that there is a difference in onset of radiation therapy between the two groups (13 days in MISS vs. 24 days in open surgery) [83]; however, this argument seems to be less important in MESCC cases, where usually an open surgery is needed for adequate decompression. ReASF has been reported to range from 9.7% [84] to 20.9% [61], with uncontrolled pain, sensory-motor deficits, and fever being the most common reasons for ReASF [84]. Analysis of the nationwide readmission database has shown that infection following surgery with sepsis and urinary infection, are the most common reasons for readmission with 37.5% of unplanned readmission in the first 90 days [85].

There are several limitations of this study. Retrospective character, low number of patients, as well as multiple surgeons involved in the surgical treatment, are clear limitations; however, this study allows you to draw conclusions on the outcome of a surgically treated cohort of patients at a single center. There is no control group of patients who underwent direct radiotherapy or radiotherapy with previous biopsy and the comparison with patients who had unstable pathological fractures without any MESCC was not performed, however total number of these patients in the defined period was too low to draw any purposeful comparisons. Further continuous multicenter prospective studies with a larger number of patients are warranted since the oncological treatment is for some cancer types evolving rapidly and prognosis and survival increase; nevertheless, surgical therapy shows promising results for quality-of-life preservation even in cases of limited prognosis. The recent establishment of prospective spine tumor registries [86] is a step in the right direction.

## 5. Conclusions

Most patients, undergoing decompression and/or stabilization for metastatic spinal tumors, have profited from surgical therapy in terms of pain and reversal of neurological deficits and overall survival. A favorable prognosis was shown in patients with a Tomita score < 7, Frankel score A–C, ECOG 0–1, and modified Tokuhashi score > 10. The complication rate is high. Modern treatment of patients with spinal metastases encompasses preservation of quality of life, promoting surgical therapy in case of pain and neurological deficits due to MESCC, even in cases of unfavorable prognosis of the disease, and the fact that 30-day mortality rates and surgical complications are high.

## Figures and Tables

**Table 1:** Sociodemographic data.

Variable		N (%) or Median (95% CI, Lower—Upper Bound)	*p*-Value
Sex	Male	104 (59.4)	0.013
	Female	71 (40.6)	
Age (years)		67 (66–70)	-
Age (categories)	<59	43 (24.6)	<0.001
	60–80	115 (65.7)	
	>81	17 (9.7)	

CI, confidence interval.

**Table 2:** Preoperative findings.

Variable		N (%)	*p*-Value
Primary neoplasm	Lung	31 (17.7)	<0.001
	Prostate	31 (17.7)	
	Breast	28 (16.0)	
	Kidney	11 (6.3)	
	Multiple myeloma	25 (14.3)	
	CUP	8 (4.6)	
	Liver	4 (2.3)	
	Pancreas	2 (1.1)	
	Melanoma	3 (1.7)	
	Gastrointestinal system (tongue, esophagus, stomach, colon, rectum)	14 (8.0)	
	Female urogenital system (ovary, uterus, vulva)	6 (3.4)	
	Other	12 (6.9)	
Affected regions	Cervical	7 (4.0)	0.024
	Cervical + thoracic	17 (9.7)	
	Lumbar	15 (8.6)	
	Sacral	1 (0.6)	
	Thoracic	38 (21.7)	
	Thoracic + lumbar	18 (10.3)	
	>2 regions	79 (45.1)	
Number of metastatic lesions	1	36 (20.6)	0.011
	2	16 (9.1)	
	3	15 (8.6)	
	4	10 (5.7)	
	5	15 (8.6)	
	>5	83 (47.4)	
Extent of tumor	Bone only	7 (4.0)	<0.001
	Epidural	165 (94.3)	
	Intradural	3 (1.7)	
Adjuvant chemotherapy	No	77 (47.2)	<0.001
	Yes	86 (52.8)	
Adjuvant radiotherapy	No	47 (28.3)	<0.001
	Yes	119 (71.7)	

**Table 3:** Clinical presentation, surgical outcome, and complications.

Variable		N (%) or Median (IQR)	*p*-Value
ECOG	Asymptomatic	7 (4.0)	<0.001
	Symptomatic but completely ambulatory	97 (55.4)	
	Symptomatic but <50% in bed during the day	27 (15.4)	
	Symptomatic but >50% in bed during the day	17 (9.7)	
	Bedbound	27 (15.4)	
Tomita predictive score (TPS)	>24 months (surgical strategy)	80 (45.7)	<0.001
	12–24 months (surgical strategy)	40 (22.9)	
	6–12 months (palliative debulking)	39 (22.3)	
	<3 months (no surgery)	16 (9.1)	
Modified Tokuhashi Score (MTS)	A (<6 months)	102 (58.3)	<0.001
	B (>6 months)	54 (30.8)	
	C (>1 year)	19 (10.9)	
Frankel score	A	5 (2.9)	<0.001
	B	3 (1.7)	
	C	31 (17.7)	
	D	67 (38.3)	
	E	69 (39.4)	
ASA classification	I	3 (1.7)	<0.001
	II	34 (19.4)	
	III	126 (72.0)	
	IV	12 (6.9)	
ASIA impairment scale	A	5 (2.9)	<0.001
	B	3 (1.7)	
	C	14 (8.0)	
	D	91 (52.0)	
	E	62 (35.4)	
Treatment effect	Deterioration	10 (5.7)	<0.001
	Improvement	122 (69.7)	
	No change	34 (19.4)	
	Not leviable	9 (5.1)	
Complications after surgery	Wound revision	10 (5.7)	<0.001
	Cardiac, respiratory, and liver failure	8 (4.6)	
	Screw malposition	14 (8.0)	
	Pulmonary embolism	3 (1.7)	
	COVID-19 infection	1 (0.6)	
	Epidural hematoma	4 (2.3)	
	CSF tearing	4 (2.3)	
	Other complications	14 (8.0)	
	Without complications	117 (66.9)	
Surgery time (minutes)		151 (105–206)	0.059
Blood loss during surgery (mL)		500 (300–800)	0.124

**Table 4:** Multivariant regression analysis for observed variables.

Variable	OR	95% CI		*p*-Value
		Lower	Upper	
ECOG (referent: ECOG ≤ 2)				
Deterioration	5.585	1.113	28.031	0.037
Improvement	1.500	0.189	11.927	0.702
No change	8.400	1.480	47.667	0.016
Tomita score (referent: <7)				
Deterioration	2.000	0.150	26.734	0.600
Improvement	1.760	0.209	14.802	0.603
No change	2.462	0.266	22.772	0.427
Frankel score (referent: A–C)				
Deterioration	1.200	0.194	7.441	0.845
Improvement	3.267	0.815	13.095	0.095
No change	3.086	0.651	14.619	0.156
Modified Tokuhashi Score (referent: ≤10)				
Deterioration	3.000	0.220	40.931	0.410
Improvement	1.700	0.320	9.036	0.534
No change	1.500	0.241	9.337	0.664
Adjuvant chemotherapy (referent: Yes)				
Deterioration	4.695	2.382	5.223	<0.001
Improvement	0.424	0.241	0.874	<0.001
No change	0.681	0.581	1.458	0.248
Adjuvant radiotherapy (referent: Yes)				
Deterioration	2.736	1.172	3.045	<0.001
Improvement	0.452	0.357	0.812	<0.001
No change	0.841	0.755	1.951	0.357

## Data Availability

The data in this study are available on request from the corresponding authors.
